# A High-Content Screening Assay for the Discovery of Novel Proteasome Inhibitors from Formosan Soft Corals

**DOI:** 10.3390/md16100395

**Published:** 2018-10-21

**Authors:** Xue-Hua Ling, Shang-Kwei Wang, Yun-Hsuan Huang, Min-Jay Huang, Chang-Yih Duh

**Affiliations:** 1 Department of Marine Biotechnology and Resources, National Sun Yat-Sen University, Kaohsiung 80441, Taiwan; cooley@mail.nsysu.edu.tw (X.-H.L.); m035020006@student.nsysu.edu.tw (Y.-H.H.); kmuhcmv@gmail.com (M.-J.H.); 2 Department of Microbiology and Immunology, Kaohsiung Medical University, Kaohsiung 80708, Taiwan

**Keywords:** proteasome inhibitor, soft coral, high-content screening, cembrane

## Abstract

The ubiquitin-proteasome system (UPS) is a major proteolytic pathway that safeguards protein homeostasis. The main 26S proteasome consists of a 20S catalytic core proteasome and a 19S substrate recognition proteasome. UPS dysfunction underlies many important clinical diseases involving inflammation, tumors, and neurodegeneration. Currently, three 20S proteasome inhibitors, bortezomib, carfilzomib, and ixazomib, have been approved for the treatment of multiple myeloma. We aim to screen UPS inhibitors for biomedical purposes. The protein interaction network of human cytomegalovirus UL76 targets UPS, resulting in aggregations of ubiquitinated proteins termed aggresomes. In this study, we demonstrated that cell-based high-content measurements of EGFP-UL76 aggresomes responded to bortezomib and MG132 treatment in a dose-dependent manner. Employing this high-content screening (HCS) assay, we screened natural compounds purified from Formosan soft corals. Four cembrane-based compounds, sarcophytonin A (**1**), sarcophytoxide (**2**), sarcophine (**3**), and laevigatol A (**4**), were found to enhance the high-content profiles of EGFP-UL76 aggresomes with relative ratios of 0.2. By comparison to the mechanistic action of proteasome inhibitors, compounds **1** and **3** modulated the accumulation of ubiquitinated proteins, with a unique pattern likely targeting 19S proteasome. We confirmed that the EGFP-UL76 aggresome-based HCS system greatly improves the efficacy and sensitivity of the identification of proteasome inhibitors.

## 1. Introduction

Soft coral reefs inhabit diverse aquatic environments worldwide, ranging from tropical to arctic/southern oceans. A soft coral reef is a sophisticated holobiont that coevolves with invertebrates, microbes, and algae. In this ecosystem, a vast number of secondary metabolites unique to soft coral species are produced [1]. The properties of these structurally novel metabolites and their roles in environmental niches have remained largely uncharacterized. Secondary metabolites are generally considered to function in defense, food capture, and interference competition beneficial to cosurvival in microenvironmental niches [2,3]. As a result, soft coral-derived metabolites exhibit diverse biological activities with therapeutic potential, such as cytotoxicity, the inhibition of inflammatory reactions, and antimicrobial and antiviral activities [4].

The ubiquitin-proteasome system (UPS) is a conserved pathway found in all three domains of life: Animals and plants, archaea, and some bacteria [5]. The UPS operates one of the vital protein degradation pathways in cells. The main 26S proteasome is a multicomplex consisting of one 20S core proteasome with peptidase activities and two subcomplexes, termed the 19S regulatory proteasome, positioned at both termini of the cylindrical 20S proteasome. Through successive ubiquitin-conjugation reactions, the target substrate is transformed into polyubiquitinated forms. The regulatory 19S proteasome coordinates the recognition and acquisition of polyubiquitinated substrates. Subsequent deubiquitination and translocation processes convey the substrates into the 20S proteasome for degradation [6,7]. Both the 19S and 20S proteasomes share conserved catalytic core structures throughout evolution and are likely regulated by common mechanisms across multiple domains of life [8,9,10]. The importance of UPS is illustrated by its involvement in almost every type of important biochemical pathway, i.e., replication, transcription/translation regulation, cellular division/differentiation, microbial infection, and immune responses [11,12]. Unsurprisingly, UPS dysfunction underlies prominent clinical diseases involving neurodegeneration, autoimmunity, and cancer [13,14,15]. The modulation of UPS activity is a promising therapeutic strategy for novel drug discovery [16]. Currently, three proteasome inhibitors have been approved by the FDA for treating multiple myeloma. Bortezomib (Velcade), carfilzomib (Kyprolis), and ixazomib (Ninlaro) inhibit the peptidase activity of the 20S proteasome, with different enzyme specificity [17,18]. To combat the drug-resistant cancer cells emerging in response to the currently available inhibitors, there is a strong demand to develop novel inhibitors targeting other subunits of the UPS.

A hallmark of UPS impairment is elevated levels of ubiquitinated proteins in the cell [15,19,20]. As a result, protein aggresomes filled with undegradable ubiquitinated substrates often appear following inhibitory events. Human cytomegalovirus (HCMV), a member of Herpesviridae, is ubiquitous in the human population and maintains asymptomatic infection in healthy people. We demonstrate that HCMV UL76 impairs proteasome function mediated by interactions with members of the UPS network, particularly S5a of the UPS [20]. S5a is present in the cytosol, and the 19S proteasome is a receptor for polyubiquitinated proteins [21]. The EGFP-UL76 aggresome features the accumulation of ubiquitinated proteins, including S5a, in the nucleus [22,23]. Interestingly, the levels of ubiquitinated proteins are synergistically enhanced in UL76-expressing cells in response to proteasome inhibitor treatment [20]. In search of novel proteasome inhibitors, we validated the feasibility of EGFP-UL76 as a cell- and imaging-based drug screening system targeting proteasome inhibition. By using proteasome inhibitors with known mechanisms of action, high-content measurements were determined for use as analytic parameters. By employing this high-content EGFP-UL76 system, we identified four cembrane-based compounds purified from *Sarcophyton trocheliophorum* and *Sarcophyton ehrenbergi* [24,25,26]. Further experiments demonstrated that these four compounds elevated the levels of polyubiquinated proteins in a dose-dependent manner comparable to the effects of bortezomib and MG132. Novel mechanisms are suggested by the finding that marine compounds **1** and **3** decreased mono- and polyubiquitinated S5a, but increased total ubiquitinated proteins, whereas the action of bortezomib and MG132 on the 20S proteasome increased all the above levels. In this study, we demonstrated the applicability of the EGFP-UL76 HCS system as an efficient and sensitive assay for the discovery of novel proteasome inhibitors.

## 2. Results

### 2.1. Validation of EGFP-UL76 High-Content Analytic Measurements by Proteasome Inhibitors

**Figure 1:**
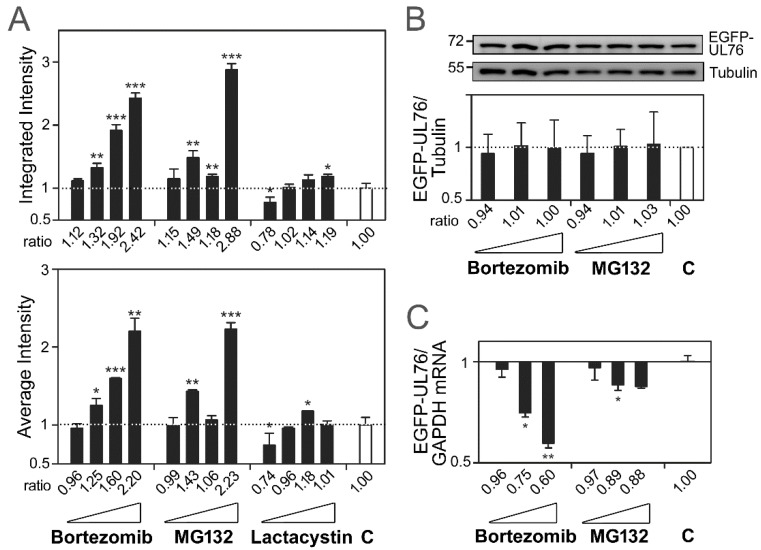
(**A**) The assessment of high-content measurements of EGFP-UL76 aggresome upon treatment with the proteasome inhibitors bortezomib (0.2, 1, 5, and 25 nM), MG132 (0.125, 0.25, 0.5, and 1 µM), and *clasto*-lactacystin β-lactone (0.08, 0.4, 2, and 10 µM). The integrated (top panel) and average (bottom panel) intensities per cell were measured, and the ratios were obtained by normalization to the control without proteasome inhibitor treatment, which is denoted by **C** throughout the text. Validation of the protein and transcript levels of EGFP-UL76 upon the addition of the proteasome inhibitors, bortezomib (1, 5, and 25 nM) and MG132 (0.25, 0.5, and 1 µM). (**B**) Western blot imaging and densitometric analyses were performed to quantitate the relative ratio of protein expression. The molecular mass markers are shown on the left in kDa. An equal level of α-tubulin was used as an internal loading control in each sample. Protein ratios of EGFP-UL76/tubulin were obtained by normalization to the control value. (**C**) Quantitative PCR was performed to assess the ratio of EGFP-UL76 mRNA expression. The transcript ratios of EGFP-UL76/GAPDH were obtained by normalization to the control value. All data points are the averages of at least three repetitive experiments. The error bars indicate standard deviations. The following symbols are used to indicate statistical significance throughout the text: * 0.01 < *p* < 0.05; ** 0.001 < *p* < 0.01; *** *p <* 0.001.

As reported in our previous publication, the HCMV EGFP-UL76 aggresome displays visible phenotypic features. The levels of polyubiquitinated proteins were not only enhanced by UL76, but also elevated by treatment with proteasome inhibitors (see Figure 4 of Lin et al. [20]). Therefore, we hypothesized that the high-content measurements of EGFP-UL76 act as a reporter for proteasome inhibition. To assess the cell system expressing EGFP-UL76 and validate the high-content measurements representing proteasome inhibition, we used bortezomib, MG132, and *clasto*-lactacystin β-lactone to establish the paradigm protocol. In this system, EGFP-UL76 was transiently expressed in HEK293T cells for two days with proteasome inhibitor treatment. Fluorescence imaging was acquired using an ImageXpress Micro Widefield HCS system. At the initial step to determine the drug dilution concentration, we tested the cytotoxicity (ED_50_) of the compounds of interest against HEK293T cells transfected with pEGFP-UL76 DNA. Both high-content nuclear count measurements and 3-(4,5-dimethylthiazol-2-yl)-2,5-diphenyltetrazolium bromide (MTT) cytotoxicity assays were used to determine the respective ED_50_ values, which were as follows (Supplementary Materials Table S1): Bortezomib, 13.1 nM and 7.76 nM; MG132, 0.97 µM and 1.65 µM; and *clasto*-lactacystin β-lactone, >10 µM and >10 µM. Next, with starting concentrations of approximately the ED_50_, the high-content screening (HCS) assay was performed using four dilutions of each tested compound. Cellular imaging was acquired after two days of transient expression of EGFP-UL76. Two software modules, Cell Scoring and Multi-Wavelength Cell Scoring, were employed for high-content measurement analyses. In total, 69 measurements were analyzed. In our configuration, an average of 12,700 nuclear counts per well without drug treatment were scored. In the initial analysis, EGFP-UL76 aggresomes with diameters above 1 µm were scored. Relative measurement ratios were calculated by comparison to the control value without treatment. As shown in Figure 1A, the integrated (top panel) and average intensity (bottom panel) of EGFP-UL76 aggresomes per cell responded to proteasome inhibitors in a dose-dependent manner. The relative ratio obtained by normalization to the control showed statistically significant increases. The integrated intensity of aggresomes per cell for treatment with 1, 5, and 25 nM bortezomib increased to relative ratios of 1.32 (*p* = 0.0056), 1.92 (*p* < 0.0010), and 2.42 (*p* < 0.0010), respectively. For 0.25, 0.5, and 1 µM MG132, the ratios were 1.49 (*p* = 0.0032), 1.18 (*p* = 0.0085), and 2.88 (*p* < 0.0010), respectively. However, *clasto*-lactacystin β-lactone exhibited a moderate impact at 10 µM, resulting in a ratio of only 1.19 (*p* = 0.0205). At diluted 0.08 µM exposure, the ratio showed a minor decrease, with a value of 0.78 (*p* = 0.0139). When the measurements were computed as the average intensity of aggresomes per cell, a similar profile was observed (Figure 1A, bottom panel). The 1, 5, and 25 nM bortezomib treatments manifested relative ratios of 1.25 (*p* = 0.0126), 1.60 (*p* < 0.0010), and 2.20 (*p* = 0.0012), respectively. The 0.25 and 1 µM MG132 treatments resulted in ratios of 1.43 (*p* = 0.0024) and 2.23 (*p* < 0.0010), respectively. Again, *clasto*-lactacystin β-lactone exhibited a meager impact, with a ratio of only 1.18 at 1 µM (*p* = 0.0373). At diluted 0.08 µM exposure, the ratio showed a minor decrease, with a value of 0.74 (*p* = 0.0217).

Before we could conclude that the elevation in aggresome intensity measurements was due to the conformational aggregation of EGFP-UL76 protein, we needed to resolve the other two mechanisms potentially involved in the enhancement of EGFP-UL76 intensity, namely, the quantities of EGFP-UL76 protein and mRNA transcript. Accordingly, both amounts were assessed by using Western blotting imaging analysis as well as quantitative PCR. Under the same cell culture and drug (bortezomib, MG132) treatment conditions, we demonstrated that the ratios of GFP-UL76 normalized to endogenous α-tubulin showed no significant difference from the control without drug exposure (*p* = 0.3346 to 0.9486) (Figure 1B). Subsequently, the ratio of EGFP-UL76 transcript was assessed by normalization to endogenous glyceraldehyde 3-phosphate dehydrogenase (GAPDH) transcript in each sample followed by comparison to the control. As shown in Figure 1C, the 1, 5, and 25 nM bortezomib treatments retained transcript ratios of 0.96 (*p* = 0.4147), 0.75 (*p* = 0.0224), and 0.60 (*p* = 0.0056), respectively. The 0.25, 0.5, and 1 µM MG132 treatments trivially decreased the transcript ratios to 0.97 (*p* = 0.1383), 0.89 (*p* = 0.047), and 0.88 (*p =* 0.1142), respectively. These results together validated that the relative ratios for EGFP-UL76 protein and transcript were not enhanced. As a result, the increases in high-content measurements in response to proteasome inhibitor treatments plausibly resulted from the redistribution and accumulation of EGFP-UL76 aggresomes that corresponded to the characteristics of proteasome inhibition.

**Figure 2:**
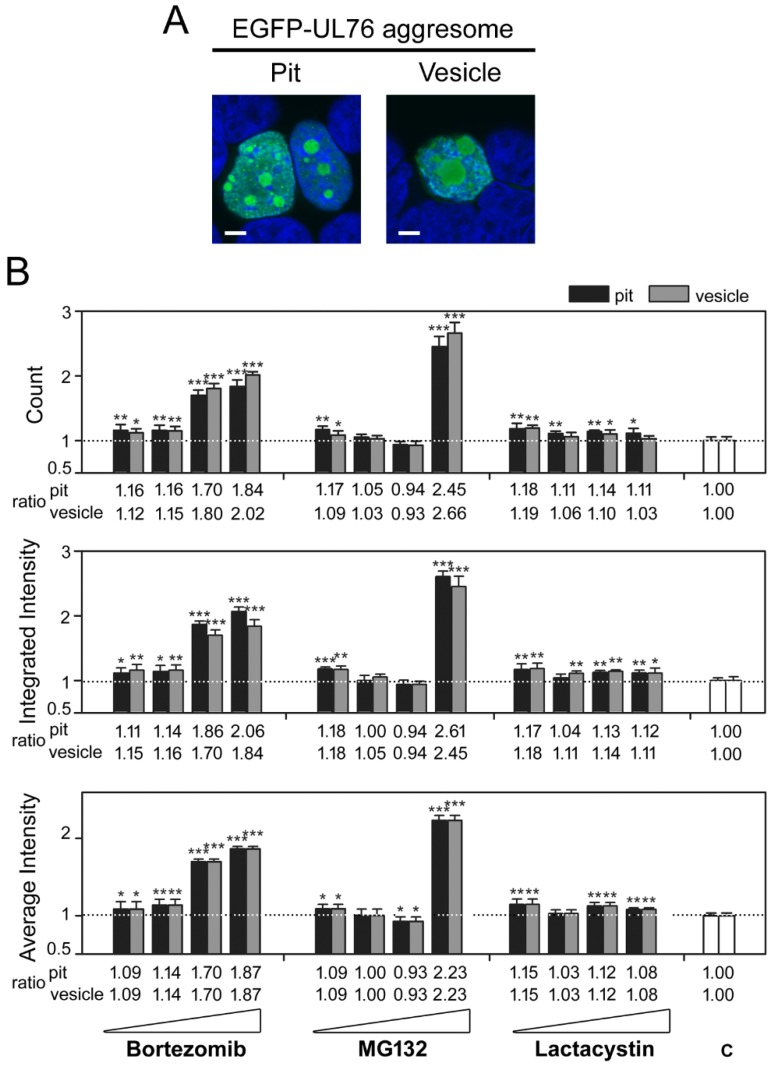
The high-content measurements of EGFP-UL76 aggresomes classified by diameter under proteasome inhibitor treatment. (**A**) Representative confocal images of EGFP-UL76 aggresomes expressed HEK293T cells transfected with pEGFP-UL76. Nuclei were counterstained with DAPI. Pit and vesicle denote aggresomes 1 to 5 µm and 5 to 20 µm in diameter, respectively. Scale bar: 5 µm. (**B**) Measurements of pit and vesicle aggresomes per cell were as follows: Count number, integrated intensity, and average intensities for pit and vesicle aggresomes, respectively. The relative ratio was normalized to the control values without inhibitor treatment. All data points are the averages of at least three repetitive experiments. The error bars indicate standard deviations. The following symbols are used to indicate statistical significance throughout the text: * 0.01 < *p* < 0.05; ** 0.001 < *p* < 0.01; *** *p* < 0.001.

To examine the phenotypic characteristics of aggresomes in detail, we classified the aggresomes by diameter into two classes, designated pit and vesicle (Figure 2A). The data shown in Figure 1 were reanalyzed accordingly. The count and the integrated and average intensities of the pit and vesicle aggresomes per cell were computed. On average, each cell had a pit count of 1.224 and a vesicle count of 0.1975 without drug treatment, indicating that EGFP-UL76 aggresomes predominantly occurred as pit sizes, ranging from 1 to 5 m in diameter, within the nucleus. Notably, among these measurements, the average intensities of pit and vesicle aggresomes were the same under the same drug treatment conditions, suggesting that the drug concentration and mechanism of action resulted in an even accumulation of EGFP-UL76 regardless of size (Figure 2B, bottom panel). In response to bortezomib treatment (Figure 2B, left set), the ratios of EGFP-UL76 aggresome measurements per cell showed stepwise increases in dose dependence and statistical significance. The count ratios per cell for pit aggresomes at 0.2, 1, 5, and 25 nM bortezomib were 1.16 (*p* = 0.0091), 1.16 (*p* = 0.0065), 1.70 (*p* < 0.0010), and 1.84 (*p* < 0.0010), respectively, whereas the ratios for vesicle aggresomes were 1.12 (*p* = 0.0170), 1.15 (*p* = 0.0067), 1.80 (*p* < 0.0010), and 2.02 (*p* < 0.0010), respectively. The ratios of integrated intensity per cell for pit aggresomes were 1.11 (*p* = 0.0171), 1.14 (*p* = 0.0114), 1.86 (*p* < 0.0010), and 2.06 (*p* < 0.0010), respectively, whereas the ratios for vesicle aggresomes were 1.15 (*p* = 0.0091), 1.16 (*p* = 0.0065), 1.70 (*p* < 0.0010), and 1.84 (*p* < 0.0010), respectively. The ratios of average intensity for pit/vesicle aggresomes were 1.09 (*p* = 0.0480), 1.14 (*p* = 0.0043), 1.70 (*p* < 0.0010), and 1.87 (*p* < 0.0010), respectively. Exposure to 0.125 and 1 µM MG132 resulted in statistically meaningful increases for all three measurements (Figure 2B, middle group). *Clasto*-lactacystin β-lactone exhibited moderate effects, with approximate ratio increases of 0.1, but this difference was statistically significant for all measurements (Figure 2B, right group).

### 2.2. Identification of Marine Natural Product Showed High-Content Characteristics of Proteasome Inhibition

**Figure 3:**
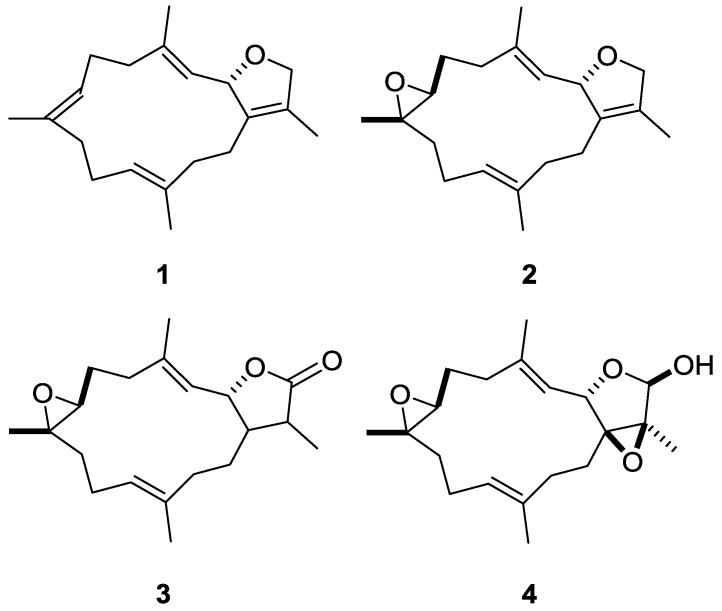
Marine natural products exhibit proteasome inhibition by high-content assays. Sarcophytonin A is (**1**), sarcophytoxide (**2**), sarcophine (**3**), and laevigatol A (**4**).

Based on the high-content measurements derived from three proteasome inhibitors, we set a threshold of an increase of 0.2 to indicate stringent proteasome inhibition for the HCS of marine natural compounds. Under this criterion, we demonstrated that the identification of four compounds exceeded the threshold and that their enhancement effects were statistically significant. Principally, their cembrane-based chemical structures have been clearly elucidated. Their purities and identities were confirmed by spectroscopic analyses (Supplementary Materials Figure S1). These marine natural products are denoted by numbers as follows: Sarcophytonin A (**1**), sarcophytoxide (**2**), sarcophine (**3**), and laevigatol A (**4**) (Figure 3) [26,27,28,29]. In addition, compounds **1**, **2**, **3**, and **4** manifested almost no cytotoxicity (ED_50_ > 25 µg/mL) in both assays (Table S1).

**Figure 4:**
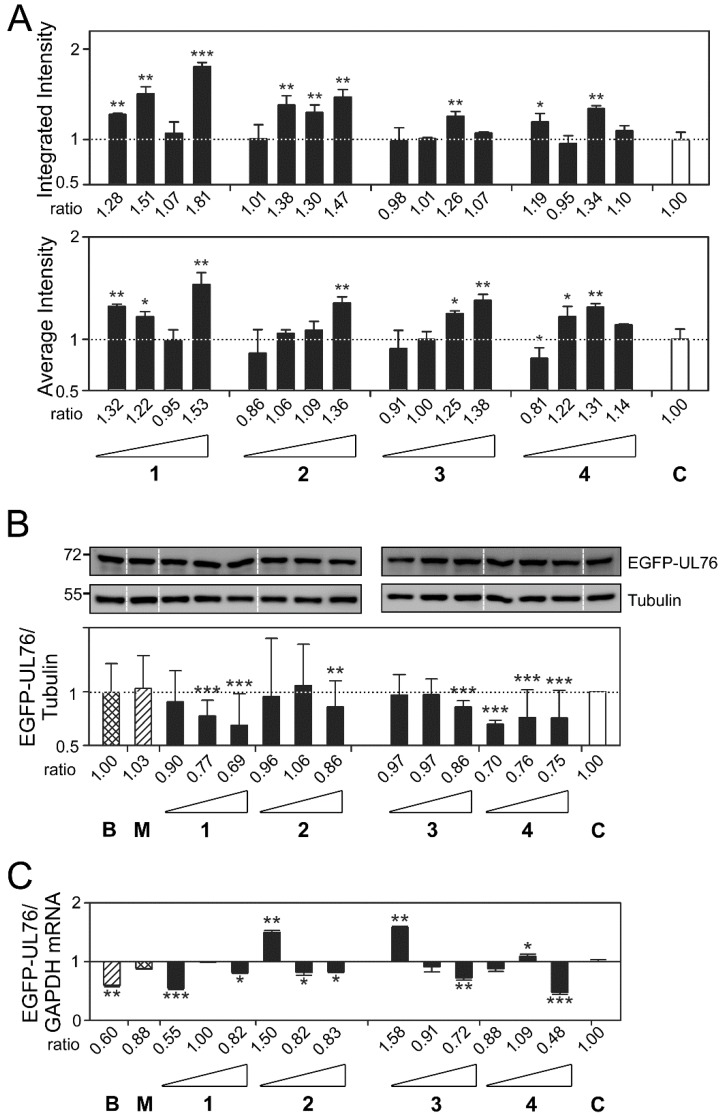
Marine compounds modulated high-content measurements of EGFP-UL76 aggresomes. (**A**) Assessment of the integrated and average intensities of EGFP-UL76 aggresomes (1 to 50 µm) per cell. The tested concentrations were 0.2, 1, 5, and 25 µg/mL for compounds **1**, **2**, **3**, and **4,** respectively. (**B**) Western blot imaging and densitometric analyses were performed to quantitate the EGFP-UL76/tubulin protein ratio with the addition of marine compound treatment at 1, 5, and 25 µg/mL. The molecular mass markers are shown on the left in kDa. (**C**) Quantitative PCR was conducted to assess the transcript ratio of EGFP-UL76/GAPDH in HEK293T cells treated with marine compounds at 1, 5, and 25 µg/mL. The high-content measurements of EGFP-UL76 with the addition of the proteasome inhibitors, bortezomib (25 nM, denoted **B**) and MG132 (1 µM, denoted **M**), were used as positive controls. All data points are the averages of at least three repetitive experiments. The error bars indicate standard deviations. The following symbols are used to indicate statistical significance throughout the text: * 0.01 < *p* < 0.05; ** 0.001 < *p* < 0.01; *** *p* < 0.001.

For the ratio of EGFP-UL76 aggresome integrated intensity per cell (Figure 4A, top panel), the highest ratios for compounds **1**, **2**, **3**, and **4** were 1.81 (*p* < 0.0010), 1.47 (*p* = 0.0020), 1.26 (*p* = 0.0085), and 1.34 (*p* = 0.0027), respectively. The highest ratios of average intensity per cell presented for compounds **1**, **2**, **3**, and **4** were 1.53 (*p* = 0.0039), 1.36 (*p* = 0.0062), 1.38 (*p* = 0.0050), and 1.31 (*p* = 0.0076), respectively (Figure 4A, bottom panel). Moreover, all these increases in ratio reached statistical significance.

Subsequently, we investigated the levels of EGFP-UL76 protein and mRNA transcript under the same experimental conditions (Figure 4B,C). In these two experiments, cells treated with bortezomib 25 nM and MG132 1 µM were used in parallel as proteasome inhibitory controls. We revealed that the ratios of EGFP-UL76/tubulin under treatment with bortezomib and MG132 showed no difference from the control level, which was consistent with the aforementioned results shown in Figure 1B. However, in cells treated with compound **1** at 1, 5, and 25 µg/mL, the ratios of EGFP-UL76/tubulin were 0.90 (*p* = 0.1033), 0.77 (*p* < 0.0010), and 0.69 *(p* <0.0010), respectively (Figure 4B). The cytotoxic ED_50_ for compound **1** was greater than 25 µg/mL in both assays (Table S1). The protein ratios for compound **2** shared the same levels as the control at the 1 and 5 µg/mL treatments, with ratios of 0.96 (*p* = 0.8907) and 1.06 (*p* = 0.9545), respectively. However, HEK293T cells treated with compound **2** at 25 µg/mL exhibited a protein ratio of 0.86 (*p* = 0.0017). Likewise, compound **3** did not affect protein ratios at 1 and 5 µg/mL. Nevertheless, a reduction to a ratio of 0.86 (*p* < 0.0010) was observed in 25 µg/mL treated cells. Compound **4** exhibited an extremely significant reduction (*p* < 0.0010) to protein ratios of 0.70, 0.76, and 0.75 at all three concentrations. The results from Western blotting analyses revealed that under 25 µg/mL treatment, neither the protein nor the mRNA ratios for EGFP-UL76/GADPH were elevated (Figure 4C). Considering these results, we postulated that the increases in EGFP-UL76 high-content measurements were likely due to protein aggregation.

**Figure 5:**
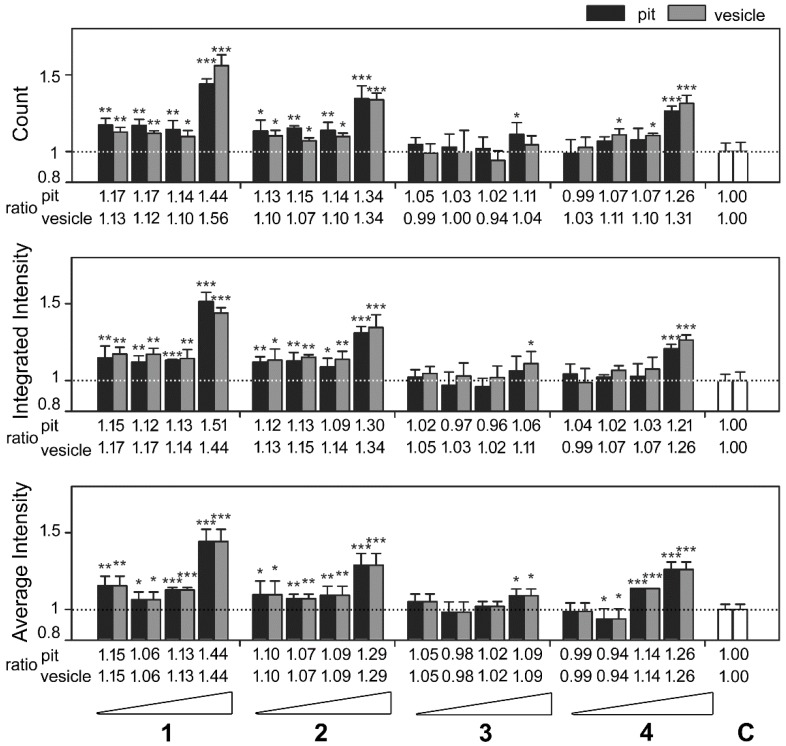
Classification of the high-content measurements of EGFP-UL76 aggresomes by size with marine compound treatment at 0.2, 1, 5, and 25 µg/mL. Pit and vesicle denote aggresomes 1 to 5 µm and 5 to 20 µm in diameter, respectively. The following pit and vesicle measurements per cell were determined: Count number, integrated intensity, and average intensity. The relative ratio was normalized to control values without marine compound treatment. All data points are the averages of at least three repetitive experiments. The error bars indicate standard deviations. The following symbols are used to indicate statistical significance throughout the text: * 0.01 < *p* < 0.05; ** 0.001 < *p* < 0.01; *** *p* < 0.001.

Extending the above analyses, we classified high-content data from Figure 4 by diameter (Figure 5) as described in Figure 2. As shown in the top panel of Figure 5, compounds **1**, **2**, **3**, and **4** at 25 µg/mL exhibited the highest ratio increases for count, as follows: For pit aggresomes, 1.44 (*p* < 0.0010), 1.34 (*p* < 0.0010), 1.11 (*p* = 0.0243), and 1.26 (*p* < 0.0010), respectively; for vesicle aggresomes, 1.56 (*p* < 0.0010), 1.34 (*p* < 0.0010), 1.04 (*p* = 0.1784), and 1.31 (*p* < 0.0010), respectively. Similar profiles were observed for the ratios of integrated intensity per cell (Figure 5, middle panel). Compounds **1**, **2**, **3**, and **4** at 25 µg/mL showed the highest ratio increases, as follows: For pit aggresomes, 1.51 (*p* < 0.0010), 1.30 (*p* < 0.0010), 1.06 (*p* = 0.1106), and 1.21 (*p* < 0.0010), respectively; for vesicle aggresomes, 1.44 (*p* < 0.0010), 1.34 (*p* < 0.0010), 1.11 (*p* = 0.0243), and 1.26 (*p* < 0.0010), respectively. The ratios of average intensity per cell were the same for pit and vesicle aggresomes (Figure 5, bottom panel). For compounds **1**, **2**, **3**, and **4**, the highest increases in ratios were observed under 25 µg/mL treatment: 1.44 (*p* < 0.0010), 1.29 (*p* < 0.0010), 1.09 (*p* = 0.0110), and 1.26 (*p* < 0.0010), respectively.

### 2.3. Cembrane-Based Compounds Exhibited the Accumulation of Ubiquitinated Proteins

**Figure 6:**
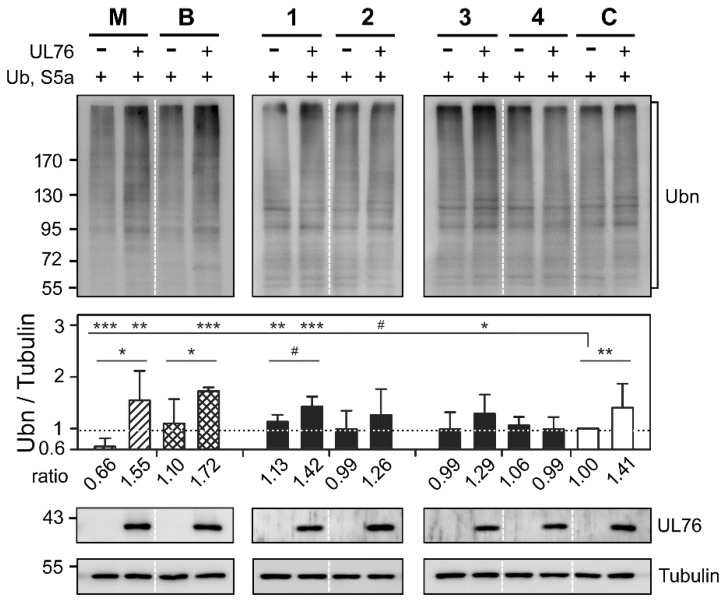
Marine compounds promote the accumulation of cellular ubiquitin-conjugated proteins. HEK293T cells transiently expressed S5a and ubiquitin proteins by transfection with pcDNA3-HA-S5a and pcDNA3-FLAG-Ub (denoted +). Cells expressing UL76 were transfected with pEF-UL76 (+), and cells not expressing UL76 were instead transfected with the cloning vector, pEF1/Myc-His C (−). Antibodies that recognized the UL76 and FLAG epitopes detected UL76 and ubiquitin-conjugated proteins, respectively. The cellular proteins were resolved in 8.5% SDS-PAGE. An equal level of α-tubulin was used as an internal loading control in each sample. The molecular mass markers are shown on the left in kDa. Cells were treated with the following drug concentrations: MG132 (M), 1 µM; bortezomib (B), 25 nM; compounds **1**, **2**, **3**, and **4**, 25 µg/mL. The intensities of the indicated polyubiquitinated area (Ubn, marked by brackets) were quantitated by densitometric analysis. Data were obtained from the average of at least three repeats. The relative ratios of ubiquitinated proteins were normalized to the values with neither UL76 production nor drug treatment. All data points are the averages of at least three repetitive experiments. The error bars indicate standard deviations. The following symbols are used to indicate statistical significance throughout the text: # 0.05 < *p* < 0.1; * 0.01 < *p* < 0.05; ** 0.001 < *p* < 0.01; *** *p* < 0.001.

**Figure 7:**
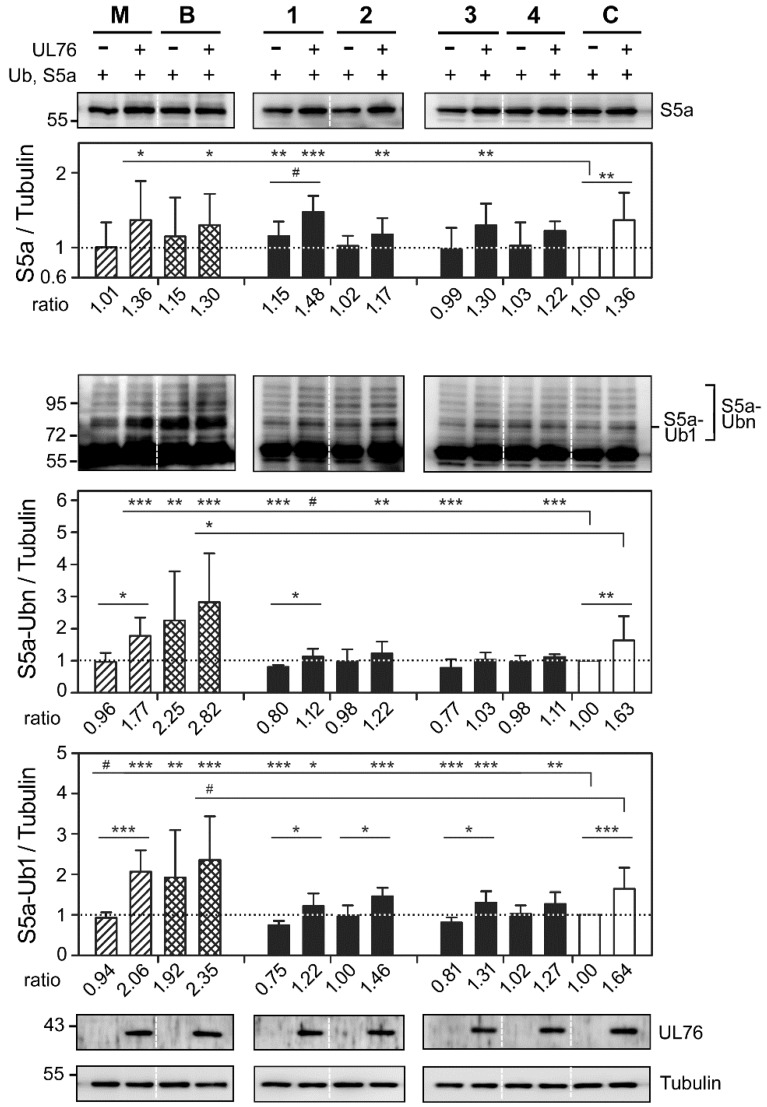
Marine compounds modulated the accumulation of cellular S5a, polyubiquitinated S5a, and monoubiquitinated S5a. HEK293T cells were transfected and exposed to the drug as described in Figure 7. S5a was detected by antibody against the HA epitope. The intensity of ubiquitinated S5a was detected by prolonging the acquisition time in the imaging detection system. The intensities of the indicated S5a, polyubiquitinated S5a (S5a-Ubn, marked by brackets), and monoubiquitinated S5a (S5a-Ub1, marked by flat bar) areas were quantitated by densitometric analyses. The ratios were normalized to the values with neither UL76 production nor drug treatment. All data points are the averages of at least three repetitive experiments. The error bars indicate standard deviations. The following symbols are used to indicate statistical significance throughout the text: # 0.05 < *p* < 0.1; * 0.01 < *p* < 0.05; ** 0.001 < *p* < 0.01; *** *p* < 0.001.

A unique hallmark of proteasome inhibition is the accumulation of undegradable polyubiquitinated proteins in cells treated with proteasome inhibitors, whether bortezomib or MG132 [7,30]. Although we demonstrated that certain marine compounds presented comparable high-content features to those of known proteasome inhibitors, two hypotheses remained to be confirmed. The first experiment aimed to investigate whether marine compounds, **1**, **2**, **3**, and **4**, elicited the accumulation of polyubiquitinated protein by comparing the impact with and without UL76 (results shown in Figure 6). The second experiment aimed to investigate whether compounds **1**, **2**, **3**, and **4** elicited the accumulation of mono- and polyubiquitinated S5a (results shown in Figure 7). In these two experiments, HEK293T cells transiently expressing S5a and ubiquitin were treated with the indicated compound. Moreover, for each compound, there were two groups of transfected cells expressing (+) or not expressing (−) UL76. As shown in Figure 6, without the influence of UL76, the ratios of ubiquitinated proteins (Ubn) under treatment with MG132, bortezomib, and compounds **1**, **2**, **3**, and **4** in comparison with the control were 0.66 (*p* < 0.0010), 1.10 (*p* = 0.4582), 1.13 (*p* = 0.0024), 0.99 (*p* = 0.9137), 0.99 (*p* = 0.8980), and 1.06 (*p* = 0.2041), respectively. Among the four marine compounds, only compound **1** enhanced the ratio of polyubiquitinated proteins in the absence of UL76. With UL76 expression, the respective ratios showed significant increases under treatment with MG132, bortezomib, and compounds **1**, **2** and **3,** with values of 1.55 (*p* = 0.0046), 1.72 (*p* < 0.0010), 1.42 (*p* < 0.0010), 1.26 (*p* = 0.0720), and 1.29 (*p* = 0.0128), respectively. These results validated that UL76 contributes to the enhancement of ubiquitinated proteins. We therefore considered whether UL76 made a dominant contribution to the final effects.

Accordingly, the ratios of ubiquitinated proteins were compared with and without the expression of UL76. The ratios for MG132 treatment were 0.66 and 1.55 (*p* = 0.0236), and the ratios for bortezomib treatment were 1.10 and 1.72 (*p* = 0.0392), with statistically significant differences. The ratio obtained by treatment with compound **1** exhibited a moderate increase from 1.13 to 1.43 (*p* = 0.0959) with additional UL76 expression. No differences were observed for compounds **2** and **3**. The ratios of ubiquitinated proteins obtained by treatment with compound **4** remained unchanged in the presence or absence of UL76. In terms of UL76 impact on the polyubiquitinated protein ratios, MG132 and bortezomib showed greater increases with UL76 expression. However, UL76 selectively enhanced the ratios of ubiquitinated proteins obtained by treatment with compound **1**, **2**, and **3**.

### 2.4. Novel Proteasome Inhibition Mechanisms

Extending our study, we examined the status of ubiquitinated S5a, which is modulated by UL76. The cell extracts and compound treatments were the same as in Figure 6. As shown in Figure 7, the ratios of S5a (top histogram), polyubiquitinated S5a (S5a-Ubn) (middle histogram), and monoubiquitinated S5a (S5a-Ub1) (lower histogram) were quantitated and compared statistically. In comparison with the control in the absence of UL76 production, the ratios of S5a for treatment with MG132, bortezomib, and compounds **1**, **2**, **3**, and **4** were 1.01 (*p* = 0.9377), 1.15 (*p* = 0.2955), 1.15 (*p* = 0.0087), 1.02 (*p* = 0.4965), 0.99 (*p* = 0.8683), and 1.03 (*p* = 0.7033), respectively. Only compound **1** increased the ratio of S5a without UL76. However, with UL76 expression, the respective ratios showed substantial increases, as treatment with MG132, bortezomib, and compounds **1**, **2,** and **3** produced ratios of 1.36 (*p* = 0.0191), 1.30 (*p* = 0.0148), 1.48 (*p* < 0.0010), 1.17 (*p* = 0.0077), and 1.30 (*p* = 0.0011), respectively. These results confirmed that UL76 contributed to the enhancement of S5a for all tested compounds except compound **4** (top histogram).

Without the influence of UL76 and in comparison with the control, the ratios of polyubiquitinated S5a for MG132 and bortezomib were 0.96 (*p* = 0.5554) and 2.25 (*p* = 0.0075), respectively, whereas the ratios for compounds **1**, **2**, **3**, and **4** were 0.80 (*p* < 0.0010), 0.98 (*p* = 0.8049), 0.77 (*p* < 0.0010), and 0.98 (*p* = 0.6376), respectively. Unexpectedly, compounds **1** and **3** showed statistically significant reductions in the ratio of polyubiquitinated S5a. Furthermore, with UL76 expression, substantial increases were observed in the respective ratios obtained by treatment with MG132, bortezomib, and compounds **1**, **2**, **3**, and **4**, which were 1.77 (*p* < 0.0010), 2.82 (*p* < 0.0010), 1.12 (*p* = 0.0697), 1.22 (*p* = 0.0023), 1.03 (*p* = 0.6119), and 1.11 (*p* < 0.0010), respectively. These results demonstrated that UL76 reversed the reductive effect in the ratios of polyubiquitinated S5a (S5a-Ubn) (middle histogram).

Based on the above results, we quantitated the ratios of monoubiquitinated S5a (S5a-Ub1), which plays a key role in the regulation of proteasome homeostasis [21]. In comparison with the control in the absence of UL76, the ratios of monoubiquitinated S5a for MG132 and bortezomib were 0.94 (*p* = 0.0927) and 1.92 (*p* = 0.0095), respectively, whereas the ratios for compounds **1**, **2**, **3**, and **4** were 0.75 (*p* < 0.0010), 1.00 (*p* = 0.9809), 0.81 (*p* < 0.0010), and 1.02 (*p* = 0.6710), respectively. Similarly, for treatment with compounds **1** and **3**, a significant reduction in the ratio of monoubiquitinated S5a was observed. With the expression of UL76, the respective ratios obtained by treatment with MG132, bortezomib, and compounds **1**, **2**, **3**, and **4** showed increases to 2.06 (*p* < 0.0010), 2.35 (*p* < 0.0010), 1.22 (*p* = 0.0151), 1.46 (*p* < 0.0010), 1.31 (*p* < 0.0010), and 1.27 (*p* < 0.0014), respectively. Again, statistical analysis indicated that UL76 significantly reversed the reductive effect on the ratio of monoubiquitinated S5a (lower histogram). This section may be divided by subheadings. It should provide a concise and precise description of the experimental results, their interpretation as well as the experimental conclusions that can be drawn.

## 3. Discussion

### 3.1. Comparison of Assay Efficacy and Sensitivity

We have hereby demonstrated not only that the EGFP-UL76 aggresome expresses a distinctive phenotype (Figure 2A), but also that the high-content measurements reflect the status of proteasome inhibition. In response to proteasome inhibition by bortezomib, MG132, and *clasto*-lactacystin β-lactone, increases in the fluorescence and intensity of EGFP-UL76 were detected (Figure 1A). Both the cellular protein and transcript levels of EGFP-UL76 remained nearly unaffected (Figure 1B,C). When the aggresomes were classified into pit and vesicle sizes, the count measurements increased in response to drug dose (Figure 2). Above all, the increasing intensities of pit and vesicle aggresomes were also associated with increasing doses of proteasome inhibitors (Figure 2B). Considering that the proteasome inhibitors, bortezomib and MG132, elicit an increase in ubiquitinated proteins (Figure 6 and Figure 7) and promote EGFP-UL76 sequestration into aggresomes (Figure 2A), we concluded that EGFP-UL76 high-content measurements correctly reflect the status of proteasome inhibition.

The experiments to validate UPS impairment were conducted mainly by Western blotting techniques. The ubiquitinated proteins were quantitated after completing a series of experiments, including SDS-PAGE, Western blotting, antigen-antibody reaction, and densitometric signal detection, followed by data analysis. The final results required labor-intensive, time-consuming experiments with high technical bias. Owing to these concerns, the protein levels are commonly described as semiquantitative.

UL76 targets many proteins in the UPS [20], and the eliciting aggresomes show a visibly spherical phenotype in high-content detection (Figure 2A). The EGFP-UL76 HCS assay revealed many advantages over the Western blotting technique. In terms of efficacy, immediately after transfection and drug treatment duration, EGFP-UL76 fluorescence acquisition was automatically conducted in the same 96-well plate used for culture. High-throughput data were streamlined to MetaExpress software modules in batch mode processing, which greatly shortened the experimental step and saved time. We have established a standard operation protocol for the HCS assay for proteasome inhibition to minimize man-made technical biases. Ultimately, we demonstrated that the EGFP-UL76 HCS assay is applicable to accelerate the screening of proteasome inhibitors.

In search of novel proteasome inhibitors from marine natural products, we successfully identified four compounds that not only exhibited comparable features to those of known UPS inhibitors, but also showed enhancement of aggresome high-content measurements (Figure 4 and Figure 5). These four cembrane-based natural compounds, sarcophytonin A (**1**), sarcophytoxide (**2**), sarcophine (**3**), and laevigatol A (**4**), were purified from the soft corals, *Sarcophyton trocheliophorum* and *Sarcophyton ehrenbergi*, in Dr. Duh’s laboratory (Figure 3 and Figure S1 in Supplementary Materials).

To our knowledge, these compounds have not been previously reported to have proteasome inhibitory activities. In reviewing the PubChem BioAssay database and literature databases, we found that sarcophytoxide (**2**) and sarcophine (**3**) stimulate alkaline phosphatase activity and increase collagen synthesis [31]. Sarcophine (**3**), a major metabolite of soft corals, exhibits a myriad of bioactivities as follows: Toxicity to fish, mice, rats, and guinea pigs [32]; inhibition of acetylcholinesterase and phosphofructokinase [32,33]; cytotoxicity against leukemia HL-60 [25]; suppression of cellular transformation [34]; inhibition of migration and wound healing [35,36]; and impairment of DNA repair by the formation of DNA adducts [37]. Laevigatol A (**4**) shows antiparasite activity [38] and anti-inflammatory activity by downregulating TNFα-induced NF-κB transcriptional activity [25].

### 3.2. Discovery of a Novel Mechanism of Action

Our HCS assay revealed a novel biological activity for compounds **1** to **4**: Impairment of protein degradation and dysregulation of S5a ubiquitination. Proteasome S5a acts as a receptor for both cytosolic and proteasome-bound ubiquitinated proteins [21]. The evidence suggests that UL76 interacts with the VWA domain of S5a, causing the elevated levels of mono- and polyubiquitinated S5a that underlie the accumulation of ubiquitinated proteins and UL76 at aggresome [20]. The characteristics of UL76 are consistent with reports that higher levels of monoubiquitinated S5a are associated with the inhibition of proteasome proteolytic activity [21,39,40].

The control inhibitor, bortezomib, inhibits the β1 caspase-, β2 trypsin-, and β5 chymotrypsin-like proteasome subunits [41,42]. Bortezomib enhances the high-content measurements of integrated and average EGFP-UL76 intensities per cell and increases the ratios of both counts and intensity for pit and vesicle aggresomes (Figure 1A and Figure 2B). Judging from Western blotting analyses, the biological activities of bortezomib were consistent with those of control UL76, showing increasing ratios of ubiquitinated proteins and specifically of both poly- and monoubiquitinated S5a (Figure 6 and Figure 7).

We considered two unexpected findings in this study to merit attention. In the absence of UL76, compound **1** modulated increasing ratios of ubiquitinated protein (Figure 6) and S5a (Figure 7, top histogram). However, compounds **1** and **3** decreased the ratios of poly-(S5a-Ubn) and monoubiquitinated S5a (S5a-Ub1) protein (Figure 7, middle and bottom histograms). These results were reversed by coexpression with UL76, which increased the ratios obtained in both high-content and Western blotting experiments. The reduction in monoubiquitinated S5a is associated with various environmental stresses, temperature shock, and cadmium exposure, resulting in increased proteasome proteolytic activity [21]. In contrast, many publications have reported that temperature shifts and metals induce protein misfolding related to proteasome dysfunction [43,44]. Dr. Prag and colleagues elaborate that S5a ubiquitination is an autoregulatory mode linked to the modulation of proteasome activity [6,40]. Taken together, these findings highlight that the mechanism of action of these cembrane-based compounds is distinct from that of bortezomib or HCMV UL76. The detailed molecular mechanisms of proteasome activity require further investigation.

### 3.3. Roles in Coral Reef Holobiont

The soft coral reef holobiont consists of symbiotic interactions among multiple organisms, encompassing eukaryotes, fungi, archaea, bacteria, and even viruses identified in recent metagenome sequencing [45]. Sarcophine (**3**), a major metabolite of soft coral species, is proposed to play a defensive role in the symbiont system [2,46]. Primarily, the proteasome is a conserved system for almost all forms of life. We suggest that the modulation of proteasome activity is likely involved in soft coral reef symbiosis.

## 4. Materials and Methods

### 4.1. Compounds

Sarcophytonin A (**1**) and laevigatol A (**4**) were prepared from the extract of soft coral *Sarcophyton trocheliophorum* following previously published protocols [24,25]. Sarcophytoxide (**2**) and sarcophine (**3**) were isolated from the soft coral *Sarcophyton ehrenbergi* as previously described [26]. To support the identity and purity of the tested compounds, ^1^H nuclear magnetic resonance (NMR) spectra are included in the Supplementary Materials (Figure S1).

### 4.2. Cell, Plasmids, and Antibodies

Human embryonic kidney large-T antigen-transformed (HEK293T) cells were grown in Dulbecco’s-modified Eagle medium supplemented with 10% fetal bovine serum (Thermo Fisher Scientific, Waltham, MA, USA). The plasmids, pEF-UL76, expressing full-length UL76 [47], and pEGFP-UL76, expressing the fusion protein, EGFP-UL76 [23], were described previously. The plasmid, pDNA3-FLAG-Ub, containing ubiquitin, was subcloned from pCGN-HA-Ub, which was provided by Dr. Jeang Kuan-Teh (National Institute of Allergy and Infectious Diseases) [20,48]. The plasmid, pcDNA3-HA-S5a, containing the full-length human S5a, was kindly provided by Yael Gus (Hebrew University of Jerusalem, Givat Ram, Israel) [20,49]. A mouse polyclonal antibody (Ab) recognizing a UL76 epitope (amino acids 244 to 267; 1:3000 dilution) was obtained as a custom Ab (Genomics BioSci & Tech., New Taipei, Taiwan) [20]. Mouse mAbs recognizing HA epitope (6E2; 1:3000 dilution) and α-tubulin (DM1A; 1:10,000 dilution) conjugated with horseradish peroxidase (HRP) were obtained from Cell Signaling Technology (Danvers, MA, USA). FLAG^®^ M2 (1:3000 dilution) mouse monoclonal Ab and secondary anti-mouse IgG-HRP Ab (1:80,000 dilution) were purchased from Sigma-Aldrich (St. Louis, MO, USA).

### 4.3. Cell-Based Screening Protocol

For high-content imaging acquisition and cell culturing, we chose a black glass-bottom 96-well PS SensoPlate™ (Greiner Bio-One, Kremsmünster, Austria). Initially, the control proteasome or marine natural products were diluted and dispensed into culture plates at the concentrations stated in the text.

HEK293T cells transiently expressing EGFP-UL76 were used in this HCS assay. HEK293T cells were seeded at 1 × 10^6^ cells onto 6-cm culture dishes one day before transfection. Then, 3 µg of plasmid DNA pEGFP-UL76 was transfected into HEK293T cells mediated by Lipofectamine Plus and Lipofectamine (Thermo Fisher Scientific, Waltham, MA, USA). After 3 h of transfection, the transfected cells were trypsinized and dispensed into black glass-bottom 96-well plates at 1 × 10^4^ cells per well in a volume of 200 µL per well, including the indicated compound at each concentration with three repeats. The culture plates containing the cells and tested compounds were incubated at 5% CO_2_ and 37 °C for 48 h. Subsequently, the cells were fixed in 1% paraformaldehyde for 10 min and simultaneously permeabilized with 0.1% IGEPAL^®^ CA-630, then stained with 1.5 µg/mL DAPI on ice for 30 min. After extensive washing with PBS, the cells were submerged in PBS, sealed in the dark, and stored at 4 °C.

### 4.4. HCS Analysis

Image acquisition was accomplished using an ImageXpress Micro Widefield HCS system (Molecular Device, San Jose, CA, USA) under an objective magnification of 20 × Ph1. Each well was acquired in 25 consecutive images in 5 × 5 sites with 38% well area coverage. Two modules of MetaExpress, Cell Scoring and Multi-Wavelength Cell Scoring, were employed to analyze the high-content measurements. Cell Scoring was configured to define nuclei marked by 4′ 6-diamidino-2-phenylindole (DAPI) staining with diameters of 8 to 15 µm, whereas EGFP-UL76 aggresomes had diameters of 1 to 50 µm. The intensity of the above background was determined according to the manufacturer’s instructions. Multi-Wavelength Cell Scoring was configured to classify aggresomes by size into pit and vesicle categories. The pit category contained aggresomes with diameters of 1 µm to 5 µm, whereas the vesicle category contained aggresomes with diameters of 5 µm to 50 µm. The data were compiled into cell-by-cell and site-by-site measurements, with a total of 69 measurements obtained for additional analysis. The relative ratio was calculated by normalization to the value of the control without drug treatment. Symbols denote the results of Student’s unpaired two-tailed *t*-tests as follows: # 0.05 < *p* < 0.1; * 0.01 < *p* < 0.05; ** 0.001 < *p* < 0.01; *** *p <* 0.001.

### 4.5. Western Blotting Imaging and Densitometric Analysis

The quantitation of the relative protein ratio by imaging was accomplished by a series of experiments, including SDS-PAGE, Western blotting, and chemiluminescent imaging techniques [50]. Cell culturing and drug treatment were performed as in the HCS assay. At the indicated drug exposure time, cells were harvested for soluble protein extraction in radio immunoprecipitation assay (RIPA) buffer (50 mM Tris, pH 7.5, 150 mM NaCl, 1% NP-40, 0.05% sodium deoxycholate, and 0.01% SDS) containing complete protease inhibitor cocktail (Roche, Basel, Switzerland). Bradford reagent (Bio-Rad, Hercules, CA, USA) was used to quantitate the soluble protein extract. As an internal control for the quantitation of a designated protein in the total soluble protein by immunoreaction, an equal level of α-tubulin was loaded in each gel well.

To achieve the initial requirement for internal normalization, 20 µg of soluble protein was loaded in each gel well for SDS-PAGE. Routinely, 10% SDS-PAGE resolves total proteins at a constant voltage of 100 V for 4 h. For the resolution of polyubiquitinated proteins, 8.5% SDS-PAGE was employed. After electrophoresis, the protein within the gel was transferred to an Immobilon membrane (Merck Millipore, Burlington, MA, USA) in Towbin buffer (48 mM Tris and 39 mM glycine (pH 9.2)) at 4 °C with a constant voltage of 30 V for 16 h. Nonspecific signals were blocked in Tris-buffered saline (TBS) (50 mM Tris and 150 mM NaCl (pH 7.5)) containing 1% skim milk (TBS-sm) for 1 h. The antibody was diluted in TBS-sm solution with gentle rocking at 4 °C for 16 h. Then, the membrane was incubated with mouse secondary antibody or tubulin mAb conjugated with HRP for 1 h at room temperature. Chemiluminescent signals were generated using an Immobilon Immunoblotting Detection Reagent (Millipore, Billerica, MA, USA). Blot imaging acquisition and data analysis were accomplished by BioSpectrum UVP Chemidoc-it 810 Imaging System VisionWorksLS (Analytik Jena US LLC, Upland, CA, USA). The configuration settings for the dynamic integration of signals acquired the enzymatic reactive chemiluminescent signals in real time when the detection reagent was added to the membrane. To calculate the relative protein ratio, the chemiluminescent emission within the linear range was used for normalization to the control value without drug treatment.

The relative ratios of internal α-tubulin were obtained by dividing by the control value without drug treatment. In the second step, the loading protein for each sample was readjusted to contain an equal ratio of α-tubulin. A second SDS-PAGE was performed on the designated proteins, and α-tubulin was detected by specific antibodies. The relative ratios were acquired and calculated. Each data point was the average of two to five repeats.

### 4.6. RNA Purification and PCR

After 48 h of transfection, HEK293T cells were harvested for RNA extraction using TRIzol^TM^ reagent (Thermo Fisher Scientific, Waltham, MA, USA). EGFP-UL76 mRNA expression was determined using real-time (RT) PCR. RNA samples were treated with DNaseI to remove DNA residues and then reverse transcribed for 120 min at 37 °C using a High-Capacity cDNA Reverse Transcription Kit (Applied Biosystems Inc., Foster City, CA, USA; cat. 4368814) according to the standard protocol of the supplier. The UL76-specific primers were as follows: Forward, 5′-CGTTCGGGCCGTTTCG-3′; reverse, 5′-GACGCCGTCCCAGATAGTC-3′. Each sample was tested in triplicate with 10 ng per 20-µL reaction volume and the forward and reverse primers at a concentration of 200 nM. Quantitative PCR was performed using the following conditions: 2 min at 50 °C, 10 min at 95 °C, and 40 cycles of 15 s at 95 °C and 1 min at 60 °C using 2_Fast SYBR green PCR Master Mix. The assays were run in an Applied Biosystems StepOne Real-Time PCR system (Applied Biosystems Inc., Foster City, CA, USA).

### 4.7. Confocal Microscopy

To visualize EGFP-UL76 confocal images, HEK293T cells were cultured on microscope cover glasses (22 × 22 mm, No. 1.5H). Cells transiently expressing EGFP-UL76 for two days were stained with DAPI under the same conditions as in the HCS assay. Confocal images were acquired using a laser scanning confocal microscope (FV1000, Olympus, Tokyo, Japan). Images were obtained by sequential excitation/emission at 405/461 nm (DAPI) and 488/510 nm (EGFP) for the fluorescence acquisition of DAPI and EGFP, respectively. Adobe Photoshop (version 6.0, Adobe Systems Inc., San Jose, CA, USA) was used to compile the images.

## Supplementary Materials

The following are available online at http://www.mdpi.com/1660-3397/16/10/395/s1, Table S1: Compound cytotoxicity assay in HEK293T cells, Figure S1: ^1^H NMR spectra for sarcophytonin A (**1**), sarcophytoxide (**2**), sarcophine (**3**), and laevigatol (**4**).
