# Supplementary material for: A High-Content Screening Assay for the Discovery of Novel Proteasome Inhibitors from Formosan Soft Corals
Source: Mar Drugs. 2018 Oct 21;16(10):395. doi: 10.3390/md16100395 (PMC6213913; doi:10.3390/md16100395)
Supplement: Supplementary file 1 [file marinedrugs-16-00395-s001.pdf]

Table 1S. Evaluation of ED_{50} cytotoxicity of tested compounds against HEK293T cells

Compound	*M* (g/mol)	Nuclear count	MTT assay
Bortezomib	384.243	13.1 nM	7.76 nM
MG132	475.630	0.97 μM	1.65 μM
Clasto-lactacystin β-lactone	213.23	> 10 μM	> 10 μM
Sarcophytonin A (**1**)	286.459	> 25 μg/ml (87.27 μM)	> 25 μg/ml
Sarcophytoxide (**2**)	302.458	> 25 μg/ml (82.66 μM)	> 25 μg/ml
Sarcophine (**3**)	316.441	> 25 μg/ml (79.00 μM)	> 25 μg/ml
Laevigatol A (**4**)	334.456	> 25 μg/ml (74.75 μM)	> 25 μg/ml

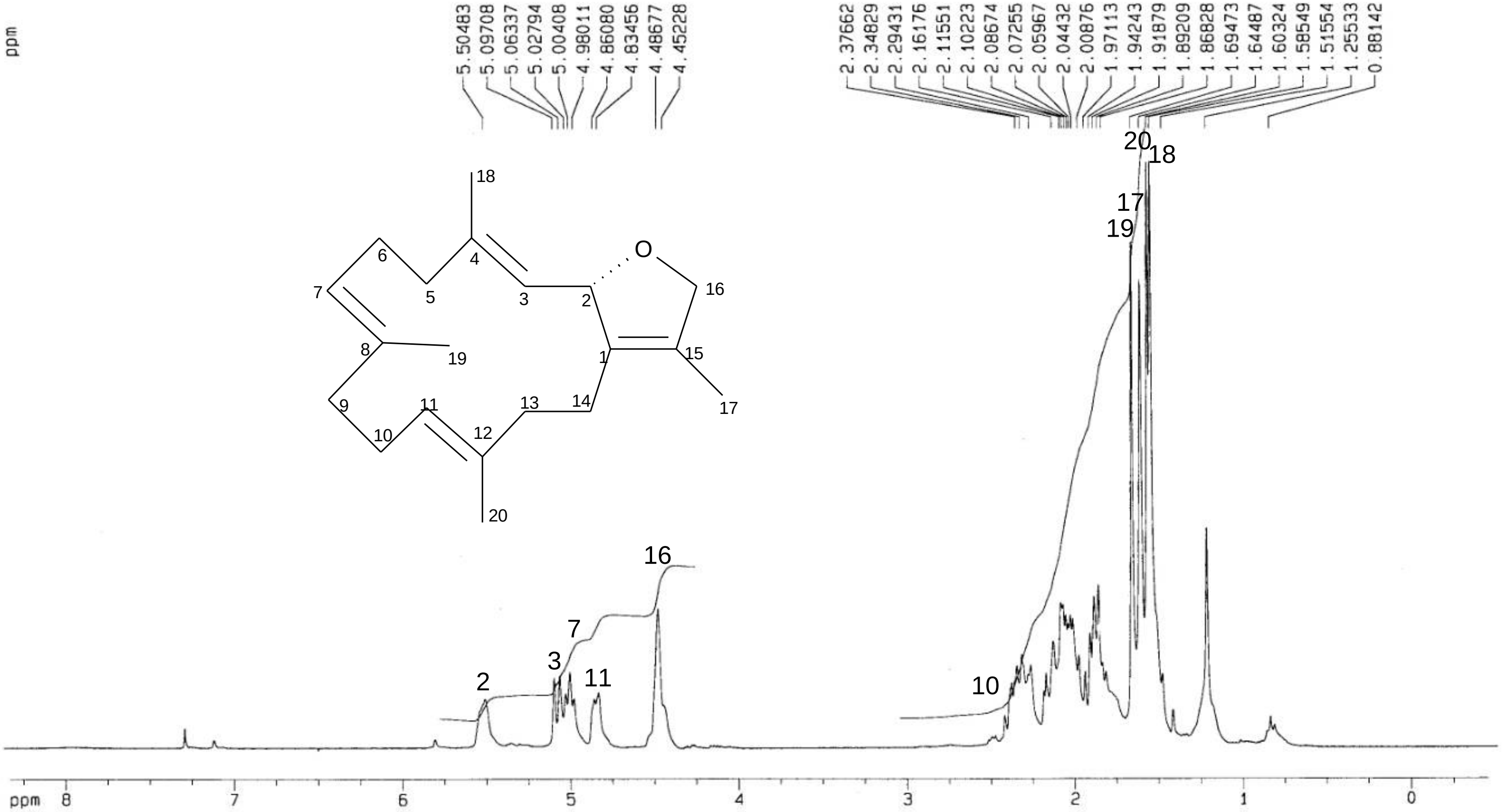

Sarcophytonin A (**1**)

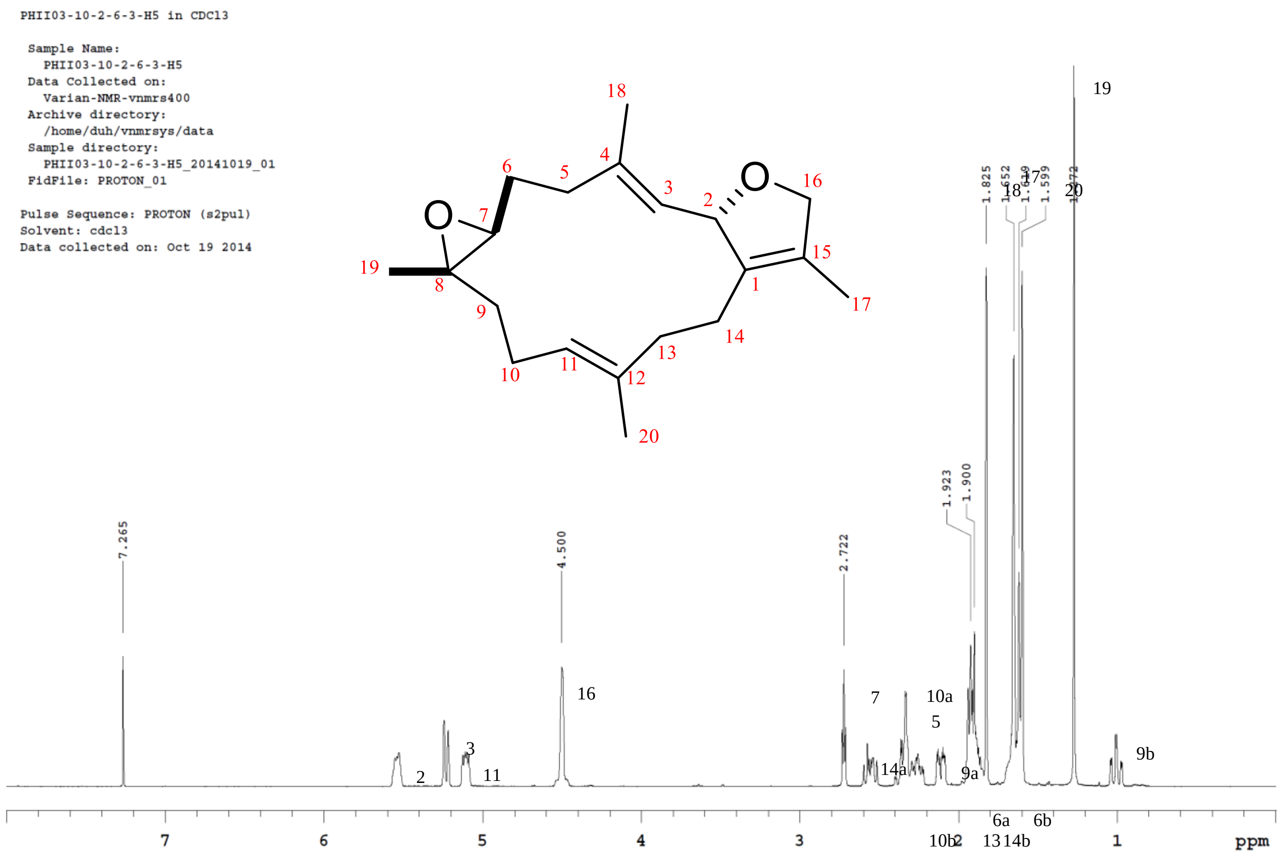

Sarcophytoxide (**2**)

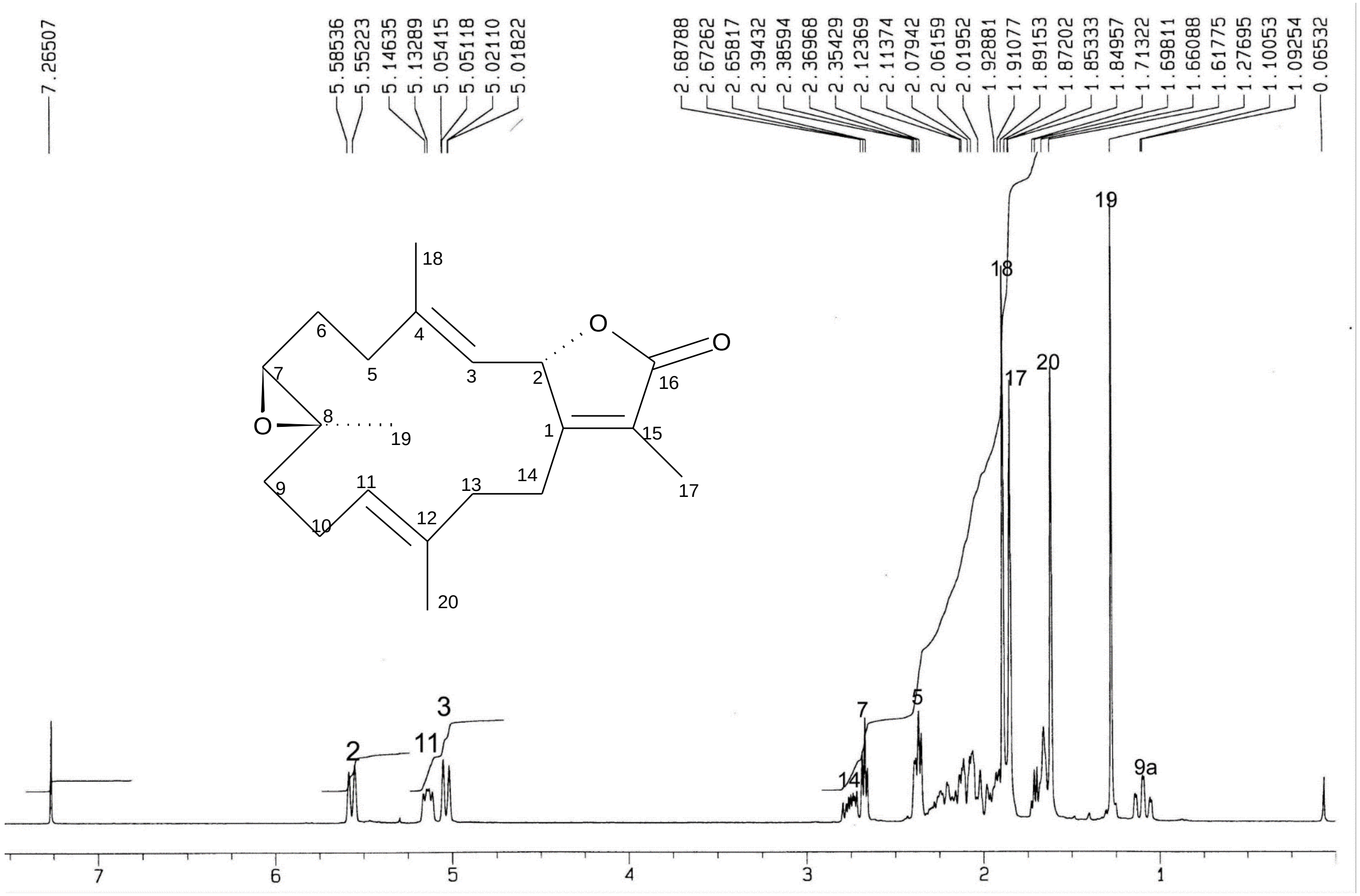

Sarcophine (**3**)

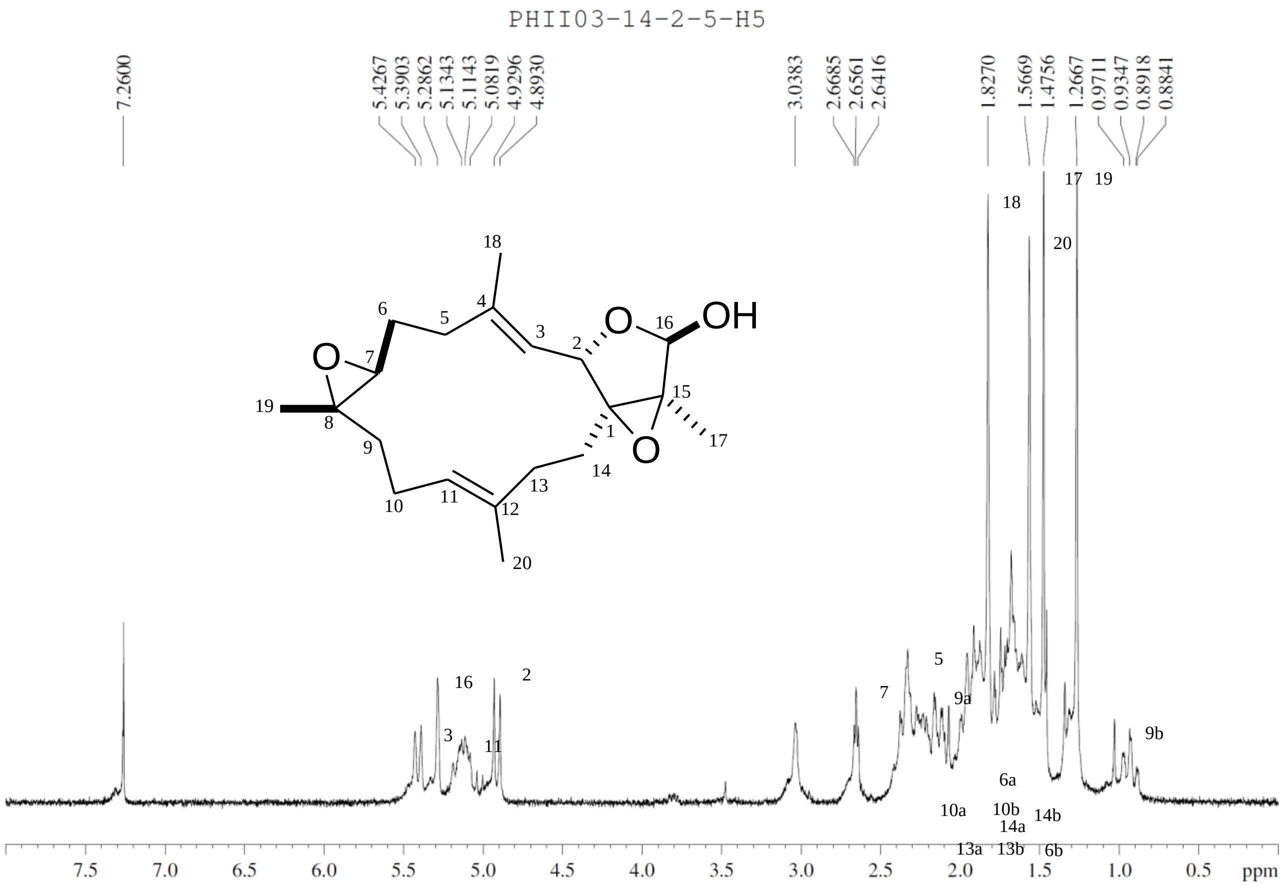

Laevigatol A (**4**)
